# A Case of REM Sleep Behavior Disorder, Narcolepsy-Cataplexy, Parkinsonism, and Rheumatoid Arthritis

**DOI:** 10.1155/2014/572931

**Published:** 2014-02-18

**Authors:** Filomena I. I. Cosentino, Angela Distefano, Giuseppe Plazzi, Carlos H. Schenck, Raffaele Ferri

**Affiliations:** ^1^Department of Neurology I.C., Sleep Research Centre, Oasi Institute for Research on Mental Retardation and Brain Aging (IRCCS), Via C. Ruggero 73, 94018 Troina, Italy; ^2^Institute of Neurological Sciences, National Research Council, 95126 Catania, Italy; ^3^Department of Biomedical and Neuromotor Sciences, University of Bologna and IRCCS Istituto delle Scienze Neurologiche di Bologna, 40123 Bologna, Italy; ^4^Department of Psychiatry, Minnesota Regional Sleep Disorders Center, Hennepin County Medical Center, University of Minnesota Medical School, Minneapolis, MN 55415, USA

## Abstract

A patient is reported in whom signs and symptoms of REM sleep behavior disorder (RBD) and narcolepsy have been associated for almost two decades with a late development of parkinsonism and rheumatoid arthritis. A 78-year-old male patient in whom RBD was first diagnosed was followed-up by clinical examination, video-polysomnography, multiple sleep latency test, cerebral magnetic resonance imaging, and dopamine transporter imaging by single-photon emission computerized tomography. The patient was found to present for almost two decades, in addition to RBD, also narcolepsy. Moreover, a late development of parkinsonism and the occurrence of rheumatoid arthritis were detected and clinically and instrumentally characterized. Patients predisposed to RBD and later parkinsonism might be susceptible to a variety of triggers that, in our patient, might have been represented by a possible latent autoimmune process leading to the development of narcolepsy with cataplexy and rheumatoid arthritis, later.

## 1. Introduction

First described in 1986 [1], rapid eye movement (REM) sleep behavior disorder (RBD) is a parasomnia characterized by repeated episodes of dream enactment behavior and REM sleep without atonia (RWA), evident during polysomnographic recording and manifested as increased phasic or tonic muscle activity on electromyogram channels [2]. RBD may be idiopathic or symptomatic and both forms are strongly associated with neurodegerative diseases; finally, RBD can be iatrogenic [3, 4]. Patients initially diagnosed with idiopathic RBD often later develop other neurological signs including parkinsonism (most frequently), orthostatic hypotension, anosmia, and cognitive impairment. Thus, RBD often heralds synucleinopathies, such as Parkinson disease, Lewy body dementia, and multiple system atrophy [5]. The time lag between the occurrence of RBD and synucleinopathy can span from one to many years or decades [6].

RBD was observed in narcoleptic patients even before its first recognition as a clinical entity by Schenck et al. [1] and was called “ambiguous sleep” [7] because of its “low phasic atonia with an extreme abundance of twitches and muscular discharges.” The prevalence of RBD in narcolepsy with cataplexy (NC) is high, being clinically evident in 45–61% of patients and polysomnographically detectable in 36–43% of them [8, 9]. Patients with NC are more frequently affected by RBD than those with narcolepsy without cataplexy [8] and in many NC patients RBD can be induced or aggravated by anticataplectic treatment (antidepressants) [10]; RBD may also be an early sign in childhood NC [11, 12]. Moreover, an increased electromyographic activity during REM sleep has been reported also in narcoleptic patients without RBD [13–15] and the prevalence of RWA, phasic electromyographic activity, and REM density is also higher in these patients than in controls [16], while patients with idiopathic RBD have a higher prevalence of RWA and a lower REM density than narcoleptic patients and controls [17]. RBD in narcolepsy also differs from the idiopathic form because of its much earlier age at onset [9, 10], different sex ratio (in the idiopathic form, RBD mostly affects men) [8–10], less violent motor behaviour, lower frequency, and different overnight distribution [18, 19].

Usually RBD emerges within a few months before narcolepsy, or concurrently with it, or after the onset of narcolepsy. To the best of our knowledge, hardly any narcolepsy case has been reported resembling the iRBD→synucleinopathy sequence, where it emerges a year or more earlier. As an example, a study on 1,152 consecutive Parkinson's disease (PD) patients reported that only 3 had a prior diagnosis of narcolepsy [20]. Even if this rate is higher than expected on general statistical grounds, the determinism of PD was suspected to be connected with the use of amphetamine in these patients rather than to the presence of prior RBD which was not reported. RBD is not mentioned also in another single case report in whom narcolepsy preceded the occurrence of PD by years [20] and in another case in whom only RWA was noticed [21]. Also, an unmedicated adolescent woman with idiopathic PD, RBD, daytime sleepiness, and REM-sleep onsets on multiple sleep latency test (MSLT) has been reported, in whom a clear diagnosis of narcolepsy was not done [22].

We report here a male patient in whom signs and symptoms of RBD and NC have been associated for years with a late development of parkinsonism and rheumatoid arthritis.

## 2. Case Report

The patient was first referred to our Sleep Research Centre in 2005, when he was 71.5 years old, because he had presented with a 12-year history of repeated nocturnal episodes of violent and automatic complex motor behaviors clearly reflecting dream enactment with frequent dream recall (war scenes or aggression). During such episodes, the patient often screamed, could fall from the bed, and could provoke lesions to the bed partner. The episodes were reported to be initially rare but had subsequently become more and more frequent with a recurrence of 3-4 times per night at the moment of our evaluation.

In addition, the patient reported infrequent episodes of weakness of the lower limbs elicited by emotions and perception of intense sounds when falling asleep, together with excessive daytime somnolence and tendency to fall asleep easily during the day and some isolated short episodes of uncontrollable but refreshing sleep, often accompanied by dream mentation. Finally, the patient also reported a mild deficit of the episodic memory.

Neurological examination was normal and no extrapyramidal signs were detected, in particular; also psychological clinical examination was normal with a score of 29 on the minimental state examination (MMSE) [23] and a score of 22 on the Epworth sleepiness scale [24]. Nocturnal laboratory polysomnography disclosed a sleep latency of 5.5 min, REM latency of 26.5 min, sleep efficiency of 85.4%, and a normal representation of the different sleep stages; periodic leg movement during sleep (PLMS) index was 47.5/hour, with a relatively low periodicity index (0.34) [25]; leg movements were more frequent but less periodic in REM sleep than in NREM sleep and did not show a clearly decreasing trend through the night. The sleep respiratory pattern was normal (apnea/hypopnea index 3.8/hour). Finally, during REM sleep an excessive amount of tonic and phasic chin EMG activations was evident with a moderate decrease of the REM sleep atonia index (0.82) [26, 27]. During the subsequent MSLT, the patient fell asleep in all of the 5 sessions with a mean sleep latency of 5 min and 36 s; no sleep-onset REM episodes were recorded.

Cerebral magnetic resonance imaging (MRI) showed a mild cortical atrophy and a small calcification of the falx. Genetic evaluation found the patient to carry the human leukocyte antigen (HLA) DQB1*0602 genotype. Thyroid hormones were within the normal limits and, among the numerous laboratory blood tests performed, a mild abnormality was found only for creatine phosphokinase (251 U/L, normal range 24–204). The patient refused the lumbar puncture procedure for the assessment of the level of hypocretin-1 in the cerebrospinal fluid.

After a careful consideration of the therapeutic possibilities with the patient and his spouse, an agreement was reached and clonazepam was started at a dosage of 0.5 mg at bedtime which was followed by an excellent and sustained beneficial effect on the nocturnal dream enactment episodes which became very rare; sleepiness was reported to be fluctuating, with short but repeated periods of worsening, interspaced by periods of lower levels of daytime somnolence. At the age of 73 years, systemic blood hypertension was diagnosed in another clinical service and an adequate and effective therapy was started and maintained chronically (telmisartan 40 mg/day). Subsequently, at the age of 76 years, the patient was referred to us again for the subjective worsening of the memory function. The psychological clinical examination disclosed the presence of a very mild short-term memory, but the MMSE score was 30. At neurological examination, a very mild hypomimic face was observed together with an olfactory deficit. The score at the Epworth sleepiness scale was 12. Electrocardiography showed sinus bradycardia and a repeat cerebral MRI study substantially confirmed the previous findings.

A new video-polysomnographic (v-PSG) recording was obtained which disclosed a sleep latency of 18.5 min, REM latency 64 min, and reduced sleep efficiency (71.5%) with a percentage of wakefulness after sleep onset of 25.2% (calculated over sleep period time); PLMS index was 2.8/hour and the sleep respiratory pattern was normal (apnea/hypopnea index 1.8/hour). Finally, during REM sleep the excessive amount of tonic and phasic chin EMG activations persisted (REM sleep atonia index 0.87).

A new careful consideration of the therapeutic possibilities was carried out and discussed with the patient and his spouse and clonazepam was continued at the same dosage of 0.5 mg at bedtime with a subsequent continuation of the excellent clinical effect on the nocturnal dream enactment episodes.

**Figure 1:**
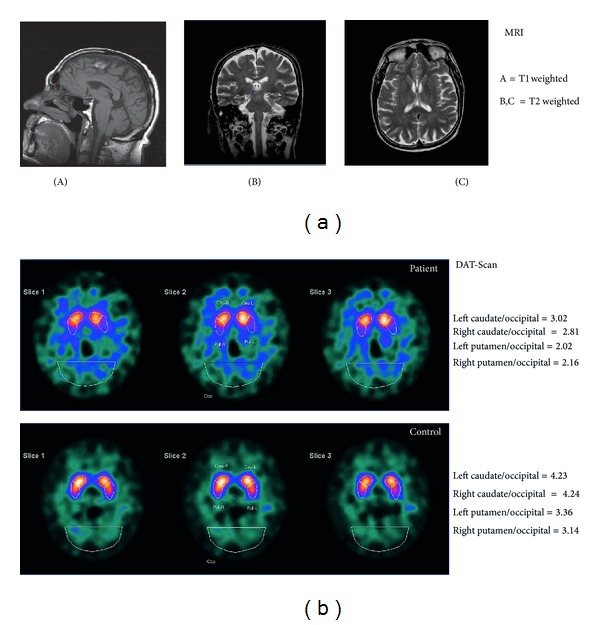
(a) Sagittal (A), coronal (B), and axial (C) magnetic resonance imaging (MRI) scans of the patient at the age of 77 years. (b) ^¹²³^FP-CIT SPECT imaging of the patient at the age of 78 years and an age- and sex-matched normal control.

The patient came back to our Sleep Research Centre at the age of 78 years because of the persistence of excessive daytime somnolence and tendency to fall asleep also in inappropriate situations. At neurological examination, hypomimic face and movement slowing were noticed again and the patient reported anosmia. MMSE score was 30. A routine electroencephalogram showed a mild slowing of the resting state alpha rhythm (7.5–8 cycles/s) and sine bradycardia was again found at electrocardiography. A third cerebral MRI again showed only a mild cortical atrophy and a small calcification of the falx (Figure 1). Nocturnal laboratory v-PSG was again performed which showed a sleep latency of 7 min, REM sleep latency of 8 min, sleep efficiency of 88.5%, and a normal representation of the different sleep stages; PLMS index was 1.8/hour and the sleep respiratory pattern was normal (apnea/hypopnea index 3/hour). During REM sleep an excessive amount of tonic and phasic chin EMG activations was still evident (REM sleep atonia index 0.86). The v-PSG was followed by a MSLT; the patient fell asleep in all of the 5 sessions with a mean sleep latency of 7 min and 12 s; no sleep-onset REM episodes were recorded. The patient gave his consent for the lumbar puncture procedure and hypocretin-1 was found to be undetectable. Dopamine transporter imaging with ^¹²³^I-2*β*-carbomethoxy-3*β*-(4-iodophenyl)-N-(3-fluoropropyl)-nortropane (^¹²³^FP-CIT) SPECT was also carried out which showed reduced striatal binding with a reduction in both the putamen and caudate nuclei to occipital cortex uptake ratios (Figure 1).

Finally, over the last four months the patient has started presenting swollen, warm, painful and stiff hand, and wrist joints; these symptoms are particularly evident in the morning on waking or following prolonged inactivity. Morning stiffness is reported to last for long time. For this problem, the patient has consulted a rheumatologist who has reached the diagnosis of rheumatoid arthritis with the additional information gathered by laboratory testing and X-rays (data not available) and a pharmacological treatment was started with methotrexate, calcium folinate, methylprednisolone, indometacin, lansoprazole, and sucralfate.

## 3. Discussion

As mentioned above, RBD usually occurs within a few months around the onset of narcolepsy and there are only rare reports of cases in whom RBD preceded the diagnosis of narcolepsy by years [28]. Differently from our patient, these cases involved young individuals in whom narcolepsy was simply not diagnosed but, most probably, was already present. In our patient, even if some signs of NC were reported to be present at the time of our first examination, the dream enactment episodes had clearly preceded the onset of excessive daytime somnolence and cataplexy. Moreover, our patient started to develop RBD at an older age (approximately 60 years), compatible with the usual age at onset of the so-called idiopathic RBD. On the other hand, the age at onset of the NC symptomatology was not typical and also the symptoms of narcolepsy were somewhat fruste or incomplete. In fact, in both MSLTs we were unable to record sleep-onset REM periods; however, REM latency was found to be abnormally reduced in two nocturnal v-PSG recordings. The patient also had the HLA DQB1*0602 genotype and undetectable levels of hypocretin-1 in the cerebrospinal fluid, which are both typical findings in NC. In parallel, also the RBD picture was typical for the classical form evolving to synucleinopathy with the onset of extrapyramidal signs several years after the onset of dream enactment episodes and the concurrent abnormalities at ^¹²³^FP-CIT SPECT [29, 30]. This form cannot be attributed to structural brain damage, as demonstrated by the repeated brain magnetic resonance imaging, or to drugs known to be able to induce RBD such as antidepressants [3, 4].

Some particular considerations should be made on the inconstant finding of increased PLMS index in our patient that was high on the first recording but was found to be within the expected range for age during the other two v-PSGs. Our findings are in agreement with the well-known night-to-night variability of PLMS [31, 32]. It should be considered here that, in the case of both RBD and narcolepsy, PLMS have been reported to be less periodic (though frequent) than those usually seen in patients with restless legs syndrome (RLS) [33, 34] and that NC patients with RLS have more periodic PLMS than those without RLS [35]. Greater amounts of PLMS have also been reported in patients with PD and RBD than in patients with PD alone; additionally, PLMS have been reported to be more numerous in iRBD patients who eventually develop PD compared to patients with iRBD who remain idiopathic after a long follow-up [36]. For these reasons, it might be concluded that an increased PLMS index, together with RBD, may be a herald of future PD [37]. However, the studies leading to this speculation were based on a single night recording that is highly influenced by the above mentioned night-to-night variability of the number of PLMS and cannot be considered to be conclusive.

In our patient we have the coexistence of two sleep disorders with a well-defined pattern of symptoms and signs, narcolepsy and RBD, which seem to constitute an association different from the usual RBD accompanying NC [17]. In fact, major behavioral episodes during REM sleep have been reported to be less frequent and violent in narcoleptic patients [9, 13], in contrast to the chin EMG abnormalities that are very often detectable, even in patients without RBD [14]. In addition, the patient developed in the last period of our observation rheumatoid arthritis that is a chronic systemic autoimmune disease characterized by inflammation of the synovial joints [38]. Interestingly, also in NC with hypocretin deficiency an autoimmune process is strongly suspected to target hypocretin cells [39].

Although we cannot rule out that the association of RBD, NC, and rheumatoid arthritis in our patient might have occurred just by chance, we should consider that the casual association between two or more unrelated clinical conditions occurs with a prevalence which is the product of the prevalence of the single conditions. Even using the highest values for the known prevalence for each condition (RBD 1/100, NC 2/1,000, and rheumatoid arthritis 1/100) we obtain that they can occur in association by chance with an extremely low prevalence of 2/10,000,000. This reinforces the idea that the association might have not been casual in our patient.

We cannot exclude that NC started early in our patient, with mild symptoms not modifying his quality of life, which was then followed by the occurrence of RBD symptoms later in life, followed by a slow evolution to PD. On the contrary, we can exclude that the NC symptoms of our patient might be regarded as the narcolepsy-like clinical picture described some years ago in PD [40] because of the undetectable levels of hypocretin-1 in the cerebrospinal fluid, a finding typical of the classical NC picture.

This rare clinical case carries five clustered, interlinked clinical disorders—three sleep disorders and two medical disorders (parkinsonism, rheumatoid arthritis)—with NC being considered to be an autoimmune disorder and rheumatoid arthritis being a recognized autoimmune disorder. Therefore, it is needed to interpret the meaning of this impressive and fascinating cluster of five clinical disorders, bearing in mind that alternative interpretations are possible. Taken all together, the considerations reported above can be synthesized in a speculative interpretation of the complex clinical picture of our patient: we can hypothesize that patients predisposed to RBD and later parkinsonism might be susceptible to a variety of triggers that, in our patient, might have been represented by a possible latent autoimmune process leading to the development of NC and rheumatoid arthritis.
